# Case report: Paratesticular dedifferentiated liposarcoma with poor prognosis

**DOI:** 10.3389/fonc.2022.1085794

**Published:** 2022-12-01

**Authors:** Hirotaka Suto, Yumiko Inui, Atsuo Okamura

**Affiliations:** ^1^ Department of Medical Oncology, The Cancer Institute Hospital of Japanese Foundation for Cancer Research, Tokyo, Japan; ^2^ Department of Medical Oncology/Haematology, Kakogawa Central City Hospital, Hyogo, Japan

**Keywords:** paratesticular dedifferentiated liposarcoma, poor prognosis, delayed diagnosis, ultrasonography, magnetic resonance imaging, well-differentiated liposarcoma

## Abstract

**Background/Aim:**

Most paratesticular liposarcomas (PLPSs) are well-differentiated liposarcomas (WDLPSs) with favourable prognoses. As such, the rare occurrence of PLPS often leads to its misdiagnosis as a hernia or hydrocele on physical examination. Curative resection of the tumour may not be possible in cases where PLPSs have transformed into dedifferentiated liposarcomas (DDLPSs) owing to a delay in diagnosis. Herein, we describe a case of unresectable paratesticular dedifferentiated liposarcoma (PDDLPS) with poor prognosis due to delayed diagnosis.

**Case Report:**

A 57-year-old man visited our hospital with a chief complaint of a right scrotal mass, which was diagnosed as scrotal hydrocele but without treatment or follow-up. Eight years later, the patient complained of abdominal distension, and a computed tomography scan revealed the presence of retroperitoneal and right scrotal masses. The right scrotal mass was removed, and histopathology revealed DDLPS. The patient was diagnosed with unresectable PDDLPS metastasising to the retroperitoneum, and the left pleura was treated with doxorubicin. After an initial response, pleural effusion and ascites increased during the sixth cycle of chemotherapy. The patient subsequently received eribulin but died 5 months after the initial DDLPS diagnosis.

**Conclusion:**

It is difficult to distinguish PLPS from benign inguinal hernia and hydrocele testis on physical examination. PLPS generally has a considerably good prognosis. However, failure to diagnose WDLPS can be dangerous as it might lead to malignant transformation to DDLPS, which has a poor prognosis. Physicians should consider this malignancy when examining patients with hernias or hydroceles of the inguinal region and should perform ultrasonography or magnetic resonance imaging.

## Introduction

Malignant soft tissue tumours usually occur in the extremities or retroperitoneum and rarely in the proximal testicular region (1, 2). The most common subtype accounting for 39%–51% of paratesticular sarcomas is liposarcoma (3–5). Most paratesticular liposarcomas (PLPSs) appear as low-growing, painless, inguinal, or scrotal masses (1, 6, 7). Therefore, they are curatively resectable and thus have a good prognosis. However, the rare occurrence of PLPS often leads to its misdiagnosis as a hernia or hydrocele on physical examination (8, 9). In some cases, due to the transformation of PLPS to dedifferentiated liposarcoma (DDLPS) because of delayed diagnosis, curative resection of the tumour may not be possible. Herein, we describe a case of unresectable paratesticular dedifferentiated liposarcoma (PDDLPS) with poor prognosis due to delayed diagnosis.

## Case description

**Figure 1:**
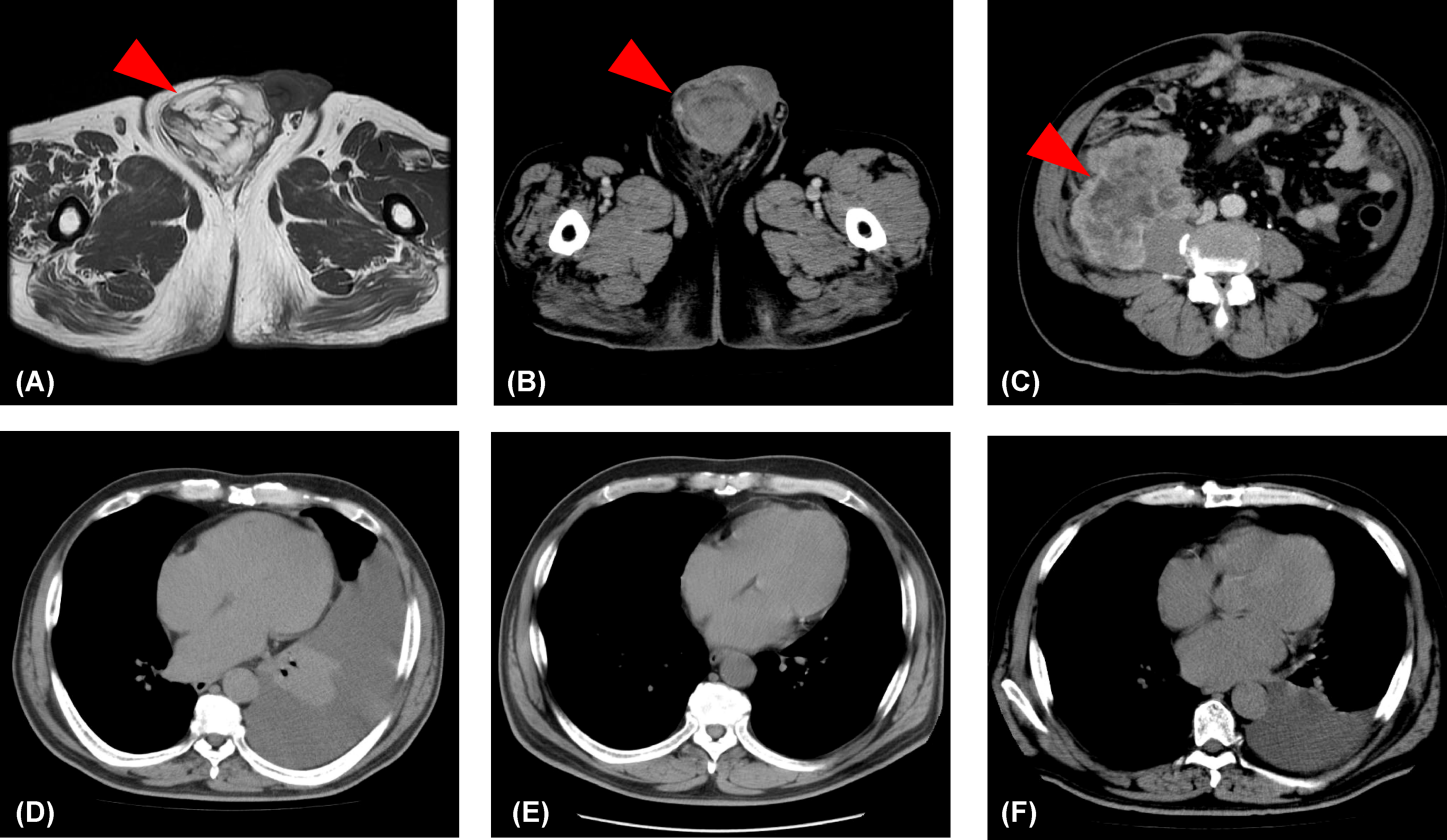
Changes in the lesions identified by imaging. **(A)** T1-weighted image showing a lipoma (red arrowhead) in the right scrotum. **(B)** An abdominal-pelvic computed tomography scan showing a substantial mass (>5 cm; red arrowhead) in the right scrotum. **(C)** An abdominal-pelvic computed tomography scan showing a mass (>10 cm; red arrowhead) in the right retroperitoneum. **(D)** A 5-week postoperative computed tomography scan showing a left pleural effusion. **(E)** A computer tomography scan showing no pleural effusion following three cycles of doxorubicin monotherapy. **(F)** A computer tomography scan showing increased pleural effusion following six cycles of doxorubicin monotherapy.

**Figure 2:**
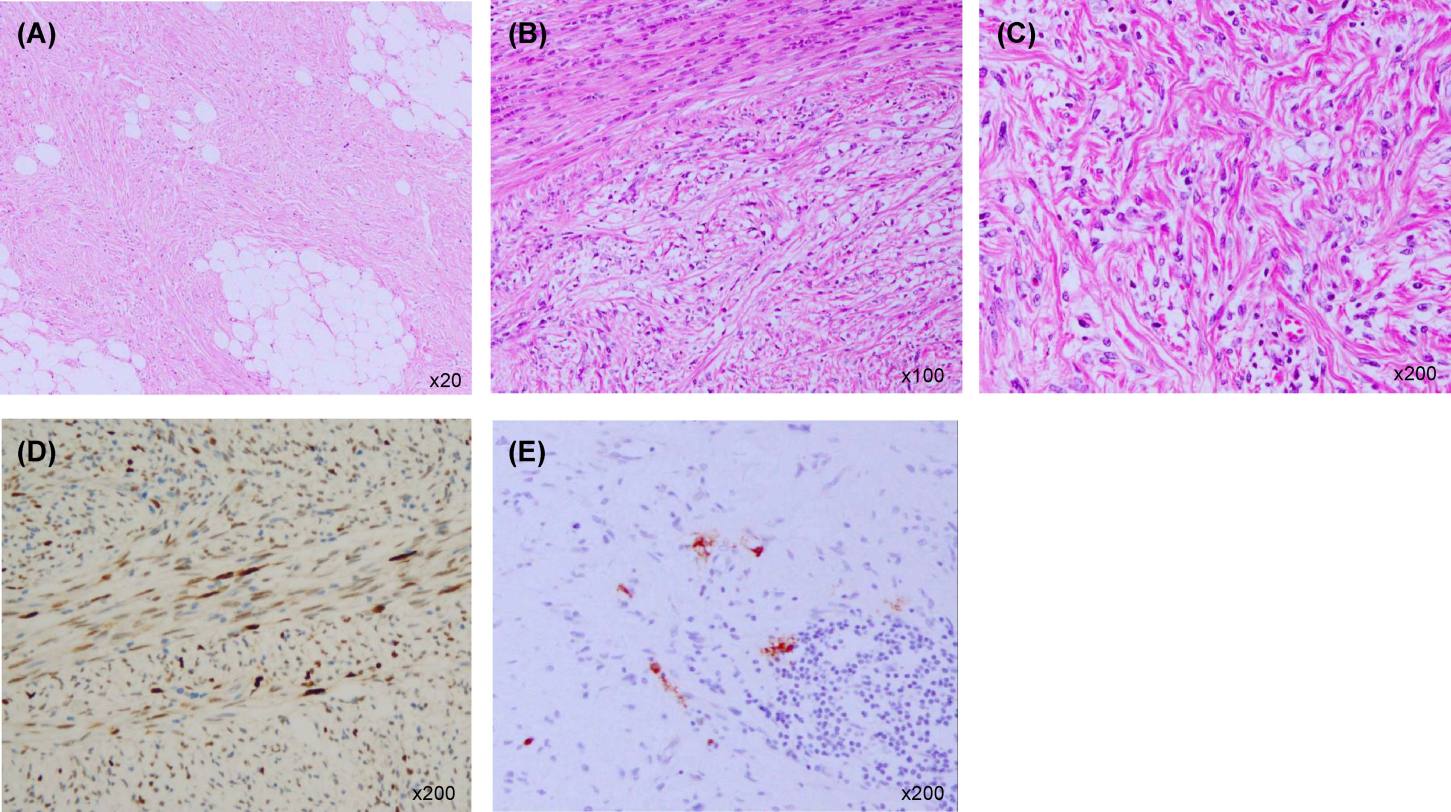
Histopathological examination of the right paratesticular mass. **(A)** Large adipocytes (haematoxylin and eosin staining ×20). **(B)** A bundle-like proliferation of medium-sized spindle-shaped cells interspersed with low-grade atypical adipoblasts (haematoxylin and eosin staining ×100). **(C)** Fibrosarcoma-like dedifferentiated liposarcoma (haematoxylin and eosin staining ×200). **(D)** Immunostaining positive for MDM2 (×200). **(E)** Immunostaining negative for S-100 protein (×200).

A 57-year-old-man presented to our hospital with the chief complaint of a painless right scrotal mass. On physical examination, his right scrotum was enlarged, but was painless, elastic, and soft. Thus, it was diagnosed as scrotal hydrops and left untreated. Two years later, a magnetic resonance imaging (MRI) scan showed a mass, 6 cm diameter, in his right scrotum with a high signal intensity on T1-weighted image. There was partial heterogeneity inside the mass, and a diagnosis of lipoma was made (**Figure 1A**). After more than 6 years without treatment, he presented again with the chief complaint of mild abdominal distention and a right scrotal mass. Palpation revealed that the scrotum was slightly firm with a smooth surface swelling. Abdominal ultrasonography revealed a rather heterogeneous, hyperechoic right scrotal mass. Abdominal-pelvic computed tomography (CT) scan showed a 50 × 70 × 68 mm3 substantial mass in the right scrotum and a 10 cm wide mass in the right retroperitoneum (**Figures 1B, C**). The peritoneum also showed nodules and ascites. There was no elevation of serum human chorionic gonadotropin or alpha-fetoprotein level, and the germ cell tumour was considered unlikely. There was a strong suspicion that the scrotal tumour was malignant. The patient underwent a diagnostic high right radical inguinal orchiectomy, which revealed a yellowish-grey-white nodule that was compressing the testis and epididymis. This nodule was 73 × 50 mm in size and well-demarcated. Histopathological studies indicated that the nodule was composed of a bundle-like proliferation of medium-sized spindle-shaped cells interspersed with low-grade atypical adipoblasts and large adipocytes (**Figures 2A–C**). Immunostaining was positive for MDM2 and negative for S-100 protein (**Figures 2D, E**). The patient was diagnosed with liposarcoma with a dedifferentiated component. We diagnosed the retroperitoneal tumour as metastasis of PDDLPS based on CT findings. Five weeks after surgery, a CT scan showed increasing ascites and left pleural effusion (**Figure 1D**). We aspirated the pleural fluid for a differential diagnosis of the left pleural effusion. The pleural effusion was haemorrhagic but showed no malignant cells. Keeping in mind the clinical presentation, we diagnosed the cause of the left pleural effusion as pleural metastasis of PDDLPS. Therefore, we initiated monotherapy with doxorubicin (DXR) for unresectable dedifferentiated liposarcoma. After three cycles of DXR monotherapy, CT showed resolution of pleural effusion (**Figure 1E**). However, CT after six cycles again showed an increased pleural effusion (**Figure 1F**). Hence, we started eribulin as the second line of treatment, but the patient did not respond and died 5 months after the definite diagnosis.

## Discussion

We report a case in which a right scrotal mass was initially misdiagnosed as a benign disease, and was later diagnosed as unresectable PDDLPS after 8 years of no treatment. The patient died 5 months after the definitive diagnosis.

More than half of PLPSs are well-differentiated liposarcomas (WDLPSs), and distant metastasis is extremely rare (1, 10, 11). However, some WDLPSs may transform into DDLPS; their rates of transformation to DDLPS are 6% in primary extremity WDLPS and 28% in paratesticular WDLPS (PWDLPS) (12). The time to dedifferentiation is 2–25 years (12). The distant metastasis rate for PDDLPS is 5%–10% higher than that for PWDLPS (1, 11, 13) (**Table 1**). Therefore, the prognosis is also worse for PDDLPS than for PWDLPS (10).

**Table 1:** Proportion of distant metastases according to tissue in paratesticular liposarcomas.

Authors	N	Histopathological type	Localised/Metastatic
Morozumi et al. (11) Montgomery et al. (1) Montgomery et al. (1) Kryvenko et al. (13)	146 19 10 42	WDLPS WDLPS DDLPS DDLPS	146 (100%)/0 (0%) 19 (100%)/0 (0%) 9 (90%)/1 (10%) 40 (95%)/2 (5%)

DDLPS, dedifferentiated liposarcoma; WDLPS, well-differentiated liposarcoma.

Liposarcomas, similar to other malignant soft tissue tumours, tend to occur in the extremities and retroperitoneum (1, 12, 14). PLPSs, in particular, are rare, accounting for 4.4% of liposarcomas (15). However, paratesticular metastases of malignant tumours are rare, and the most common primary site of metastasis is from solid tumours, such as gastric cancer (16). Paratesticular metastasis of primary retroperitoneal DDLPS is extremely rare (1).

This case showed not only a dedifferentiated component within the paratesticular tumour but also a highly differentiated component. Therefore, it is quite plausible that the PWDLPS transformed into PDDLPS and metastasised to the retroperitoneum and pleura in this patient.

First-line chemotherapy regimens containing DXR are recommended for liposarcomas with distant metastases. The response rate of DDLPS is 24%, the median progression-free survival (PFS) is 4 months, and the median overall survival (OS) is 25 months (17). Although eribulin is effective as a second-line treatment for liposarcoma, the response rate in DDLPS is 0%, the median PFS is 2 months, and the median OS is 8 months (18). Thus, there are limited effective treatments for dedifferentiated liposarcoma, including second-line chemotherapy. In our case, pleural effusion was resolved after DXR administration but worsened after approximately 4 months and did not respond to eribulin; the patient died only 5 months after diagnosis.

On physical examination, it is difficult to distinguish PLPS from benign inguinal hernia and hydrocele testis; however, PLPS has a considerably better prognosis as most PLPSs are WDLPSs with no possibility of metastasis and possible surgical resection. However, failure to timely diagnose WDLPS can be dangerous and may lead to malignant transformation into DDLPS, which has a poor prognosis. Ultrasonography shows a uniform hypoechoic area in the case of hydrocele testis but a heterogeneous hyperechoic area in the case of liposarcoma. In addition, MRI shows high signal intensity on T1-weighted images in the case of a fat-fitting inguinal hernia and lipoma and a low signal intensity in the case of liposarcoma. Hence, physicians should consider this malignancy when examining patients with hernias or hydroceles of the inguinal region and should perform ultrasonography or MRI.

## Data availability statement

The original contributions presented in the study are included in the article. Further inquiries can be directed to the corresponding author.

## Ethics statement

The studies involving human participants were reviewed and approved by The Institutional Review Board Kakogawa Central City Hospital Ethics Committee. The patient’s family provided their written informed consent to participate in this study.

## Author contributions

Conceptualisation: HS, and AO. Methodology: HS, and AO. Investigation: HS, YI, and AO. Data curation: HS, YI, and AO. Writing—original draft preparation: HS. Writing—review and editing: HS, YI, and AO. Supervision: AO. All authors contributed to the article and approved the submitted version.

## Acknowledgments

The authors would like to thank Editage (www.editage.com) for English language editing.

## Conflict of interest

The authors declare that the research was conducted in the absence of any commercial or financial relationships that could be construed as a potential conflict of interest.

## Publisher’s note

All claims expressed in this article are solely those of the authors and do not necessarily represent those of their affiliated organizations, or those of the publisher, the editors and the reviewers. Any product that may be evaluated in this article, or claim that may be made by its manufacturer, is not guaranteed or endorsed by the publisher.
